# Content validation of a questionnaire on healthcare personnel's perceptions of technologies

**DOI:** 10.15649/cuidarte.4145

**Published:** 2024-12-19

**Authors:** Maritza Díaz Rincón, Paula Constanza Arango Franco, Jose Alejandro Vergel Torrado, Olga Lucia Lora Díaz

**Affiliations:** 1 Corporación Universitaria Minuto de Dios-UNIMINUTO, Bogotá, Colombia. maritza.diaz.r@uniminuto.edu.co Corporación Universitaria Minuto de Dios-UNIMINUTO Corporación Universitaria Minuto de Dios-UNIMINUTO Bogotá Colombia maritza.diaz.r@uniminuto.edu.co; 2 Corporación Universitaria Minuto de Dios-UNIMINUTO, Bogotá, Colombia. paula.arango-f@uniminuto.edu.co Corporación Universitaria Minuto de Dios-UNIMINUTO Corporación Universitaria Minuto de Dios-UNIMINUTO Bogotá Colombia paula.arango-f@uniminuto.edu.co; 3 Corporación Universitaria Minuto de Dios-UNIMINUTO, Bogotá, Colombia. jose.vergel-t@uniminuto.edu.co Corporación Universitaria Minuto de Dios-UNIMINUTO Corporación Universitaria Minuto de Dios-UNIMINUTO Bogotá Colombia jose.vergel-t@uniminuto.edu.co; 4 Universidad del Sinú Elías Bechara Zainúm-UNISINÚ, Cartagena, Colombia. olora@unisinucartagena.edu.co Universidad del Sinú Elías Bechara Zainúm-UNISINÚ Universidad del Sinú Elías Bechara Zainúm-UNISINÚ Cartagena Colombia olora@unisinucartagena.edu.co

**Keywords:** Digital Technology, Health Personnel, Health Knowledge, Attitudes, Practice, Surveys and Questionnaires, Validation Study, Tecnología Digital, Personal de Salud, Conocimientos, Actitudes y Prácticas en Salud, Encuestas y Cuestionarios, Estudio de Validación, Tecnologia Digital, Pessoal de Saúde, Conhecimentos, Atitudes e Prática em Saúde, Inquéritos e Questionários, Estudo de Validação

## Abstract

**Introduction:**

Across the world, multiple institutions in the health sector actively promote the adoption and expansion of health technology innovations, driven by their potential benefits in improving medical care quality. The successful integration of health technologies into healthcare settings brings significant changes to work activities and depends, in part, on their acceptance and appropriation by healthcare personnel.

**Objective:**

To determine the content validity of a questionnaire adapted to assess perceptions and attitudes toward health technologies.

**Materials and Methods:**

Content validity was assessed through expert judgment using the model proposed by Escobar and Cuervo (2008). A 28-item questionnaire was adapted to assess health personnel's perceptions and attitudes toward technologies, and content validity was determined using Aiken's V coefficient. The Brennan and Prediger coefficient was used to assess agreement among experts.

**Results:**

The Aiken V coefficient was 0.98 (95% CI: 0.88 - 1.00) for the entire instrument. The expert agreement was almost perfect.

**Discussion:**

Most of the studies evaluating perceptions and attitudes toward technologies do not include validation through expert judgment before conducting statistical validation.

**Conclusion:**

According to the criteria of the consulted experts, the questionnaire's content validity is acceptable for assessing perceptions and attitudes toward health technologies.

## Resumen

**Introducción:**

En el mundo, múltiples instituciones en el campo de la salud promueven activamente la adopción y la expansión de innovaciones en tecnologías sanitarias, motivadas por potenciales beneficios en la mejora de la calidad de la atención médica. La integración exitosa de las tecnologías en el ámbito sanitario trae consigo cambios significativos en las actividades laborales que en parte depende de la aceptación y apropiación de éstas por parte del personal de salud.

**Objetivo:**

Determinar la validez de contenido del cuestionario adaptado para evaluar las percepciones y actitudes hacia las tecnologías sanitarias.

**Materiales y métodos:**

Una validez de contenido fue llevada mediante el juicio de expertos, utilizando el modelo propuesto por Escobar y Cuervo (2008). Se adaptó un cuestionario de 28 preguntas para evaluar percepciones y actitudes del personal de salud hacia las tecnologías y se determinó la validez de contenido mediante el método V de Aiken. La prueba estadística de Brennan y Prediger se empleó para evaluar la concordancia entre los expertos.

**Resultados:**

Se obtuvo un índice de consistencia V de Aiken de 0,98 (IC 95%: 0,88 - 1,00) para todo el instrumento. La concordancia entre los expertos fue casi perfecta.

**Discusión:**

Gran parte de los estudios donde se han evaluado percepciones y actitudes hacia las tecnologías, no se aplica el proceso de validación por juicio de expertos previo a la validación estadística.

**Conclusiones:**

El cuestionario posee una validez de contenido aceptable para evaluar percepciones y actitudes hacia las tecnologías sanitarias, según el criterio de los expertos consultados.

## Resumo

**Introdução:**

Em todo o mundo, múltiplas instituições da área da saúde promovem ativamente a adoção e expansão de inovações em tecnologias de saúde, motivadas por potenciais benefícios na melhoria da qualidade dos cuidados médicos. A integração bem-sucedida de tecnologias na área da saúde traz consigo mudanças significativas nas atividades de trabalho que dependem em parte da aceitação e apropriação destas por parte dos profissionais de saúde.

**Objetivo:**

Determinar a validade de conteúdo do questionário adaptado para avaliar percepções e atitudes em relação às tecnologias em saúde.

**Materiais e métodos:**

A validade de conteúdo foi realizada por meio de julgamento de especialistas, utilizando o modelo proposto por Escobar e Cuervo (2008). Um questionário de 28 questões foi adaptado para avaliar as percepções e atitudes do pessoal de saúde em relação às tecnologias e a validade de conteúdo foi determinada pelo método V de Aiken. O teste estatístico Brennan e Prediger foi utilizado para avaliar a concordância entre os especialistas.

**Resultados:**

Obteve-se índice de consistência Aiken V de 0,98 (IC 95%: 0,88 - 1,00) para todo o instrumento. O acordo entre os especialistas foi quase perfeito.

**Discussão:**

Muitos dos estudos onde foram avaliadas as percepções e atitudes em relação às tecnologias não aplicam o processo de validação por parecer de especialistas antes da validação estatística.

**Conclusões:**

O questionário possui validade de conteúdo aceitável para avaliar percepções e atitudes em relação às tecnologias em saúde, segundo critérios dos especialistas consultados.

## Introduction

The integration of technologies in healthcare aims to provide high-quality services and promote the efficient use of available resources^1^. Technologies encompass the set of resources and strategies used to respond to health needs, both individual and collective, in healthy or sick people, including a range of tools and solutions^2^. Several international organizations, such as the World Health Organization (WHO), the Pan American Health Organization (PAHO)^3^, the Organization for Economic Cooperation and Development (OECD)^4^, the World Bank, and the Inter-American Development Bank (IDB)^5^, actively support the implementation and expansion of health technology innovations worldwide^6^. However, investments not only involve risks but also demand a dynamic understanding of technological culture, organizational structures, and institutional adjustments within the parameters of the regulatory framework^7^. These interests are driven by the potential benefits of implementing technologies, including reduced direct and indirect health system costs, enhanced quality of care^8^, greater diagnostic accuracy and efficacy, real-time patient monitoring, improved chronic disease management, and increased administrative efficiency, among other benefits^9^. However, without an awareness and understanding of the potential benefits and changes that technology can bring to healthcare, healthcare workers may be hesitant to adopt them. The transition to digital is far from easy, certain, or predictable and is likely to be disruptive or transformational, with lasting effects on organizational outcomes, including technical capabilities and behaviors^7^. 

Figures published by the OECD in 2019 describe some of the organizational consequences of implementing technologies, among which a "greater demand for cognitive and non-cognitive competencies of personnel" stands out^10^, taking into account that human resources are ultimately responsible for technology implementation, which in turn depends on personal skills and adaptability to the specific needs of the context^11^. It is important to emphasize that socio-cultural factors play a fundamental role in health personnel' attitudes toward adopting or rejecting health technologies, which directly impacts the effectiveness of their implementation and use^12^. Although many technologies have shown their capacity to enhance both diagnosis and treatment, technology assimilation and integration into practice have been slow. Technology reluctance can be attributed to several factors, including the learning curve associated with using new technologies, potential communication limitations, the transmission of information through technology applications, privacy and security concerns, the need for fully integrated health information systems, ease of use, cost, familiarity with the technology, and perceived productivity benefits, among others^13^. 

Having tools that transcend disciplines is essential to address perceptions and attitudes toward health technologies in the work context, as well as providing information to design more effective, user-centered implementation strategies that ensure the sustainability of health technology interventions. Therefore, this study aims to determine the content validity through expert judgment of a questionnaire adapted to assess health personnel's perceptions of and attitudes toward health technologies.

## Materials and Methods

Content validation through expert judgment was conducted using the methodology proposed by Escobar and Cuervo (2008). This approach involves defining the objective of the evaluation, selecting the judges, explaining the questionnaire's categories and indicators, designing the evaluation grid, and analyzing the data provided by the experts^14^. 

For instrument adaptation, previous scientific literature on the assessment of perceptions and attitudes toward information and communication technologies (ICTs) among health personnel^15^–^22^, diagnostic and therapeutic, were considered, as well as the WHO guide for developing knowledge, attitude, and practice surveys, which outlines six steps: a) defining the survey objectives b) developing the survey protocol c) adapting the questionnaire d) conducting the survey e) analyzing the data 6) using the data^23^. Only the first three steps were performed for the purposes of this research.

**Participants**

Content validation was conducted through expert judgment. Before selecting the experts, the required profile was defined. This profile included experience in instrument validation, being a health professional with a strong academic or professional background, or expertise in other fields with training and experience in technologies, considering the interdisciplinary nature of the constructs being evaluated. E-mail was the means of communication with the judges during the validation process. The experts were recruited from health and education institutions and invited based on an evaluation of eligibility criteria. Subsequently, informed consent form was provided. Once the experts voluntarily agreed to participate in the study and completed the informed consent form, they were provided with the questionnaire, the evaluation instrument, instructions, a survey to collect personal and academic information, and the protocol summary. 

**Instruments**

A validation instrument was adapted containing the questionnaire items distributed in the categories of sufficiency, clarity, coherence, and relevance, as proposed by Escobar and Cuervo (2008)^14^. Each category had four rating levels on a Likert-type scale. An additional column was included for the experts' comments, if any. 

**Data analysis**

Aiken's V coefficient^24^ was used to assess the content validity of the questionnaire. This coefficient, which varies between 0 and 1, allows us to measure the items' relevance to the content domain, considering the judges' ratings^25^. However, the algebraically modified equation of Penfield and Giacobbi was used to measure the degree of agreement among the experts^26^. 




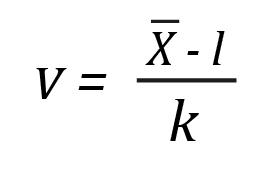




In Equation 1, X ® represents the sample mean of the judge’s ratings, l indicates the lowest possible rating, and k represents the difference between the maximum rating and the minimum rating. 

Considering that sampling error affects V, a more precise estimate of this parameter was obtained by calculating the confidence interval (CI) using Wilson's 1927 score method to determine the range of possible ratings. This method is asymmetric, exact, and does not require the assumption of normality in the variable's distribution^27^. Likewise, it was confirmed that the coefficient value was greater than the established cut-off point, with V ≥ 0.80 being deemed acceptable^28^. Finally, a lower limit of 0.7 and an upper limit of 1.0 for the confidence interval were set as item retention criteria^29^. Confidence intervals that include a value of 1.0 indicate high inter-rater consistency. 




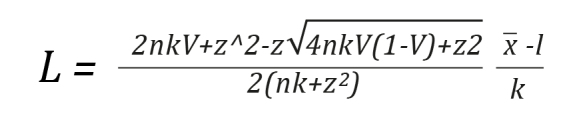







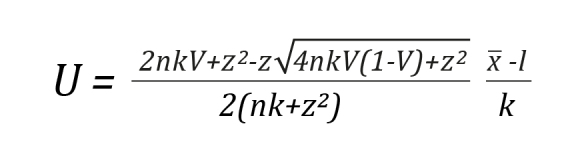




In Equation 2, L represents the lower limit of the interval, and in Equation 3, U represents the upper limit of the interval. The Z corresponds to the value of a standard normal distribution, V represents Aiken's coefficient calculated by Equation 1, and n is the total number of judges involved. The entire content validation data is available for free access and consultation in Harvard Dataverse^30^. 

Finally, the Brennan and Prediger 1981 statistical test, useful for assessing agreement among multiple raters and categories, was used to evaluate agreement among expert judges. Agreement level was considered low with a score <0.00, fair between 0.00 and 0.20, moderate between 0.41 and 0.60, substantial between 0.61 and 0.80, and almost perfect between 0.81 and 1.00^31^,^32^. Analyses were performed using STATA® version 16 statistical software and Microsoft® Excel. 

**Ethical considerations**

In compliance with Colombian Resolution 08430 of 1993, which outlines scientific standards for health research, the study was considered as no-risk, as it involved no interventions or behavior modifications^33^. The ethical principles of the Declaration of Helsinki were also observed. All participants signed an informed consent form, which was sent to them by email. The informed consent detailed the confidentiality and anonymity of their identities and the conditions of their participation in the study. 

## Results

A 28-item questionnaire was validated, consisting of 7 questions on work-related aspects and 21 questions on perceptions and attitudes toward health technologies. The questions were based on the modified Technology Acceptance Model (TAM) questionnaire^16^, the Technology Acceptance in Health Systems (ACEPTESS) questionnaire^34^, and additional questions we formulated (Table 1). Seven experts participated in the content evaluation, including four health professionals and three from other disciplines. Among the experts, two held postdoctoral degrees, two had PhDs, one had a master's degree, and two had medical specialties. All the experts had experience and/or training in instrument validation. 

Regarding the overall content validation of the questionnaire, it yielded an Aiken's V consistency index of 0.98 (95% CI: 0.88–1.00). Likewise, all items received favorable ratings across the four evaluated categories, with V values exceeding 0.80, which is considered adequate. While no items required elimination from the questionnaire, it was noted that items 11 and 12 had lower confidence interval limits that were closest to the established critical threshold. Table 2 presents Aiken's V analysis results for each item, distributed in four categories. 

**Table 1:** Questionnaire items

Dimension	Item	Response options
Work information	1. Profession/ occupation:	(1) Nursing assistant (2) Nurse (3) Physician (4) Nutritionist (5) Bacteriologist (6) Microbiologist (7) Occupational therapist (8) Respiratory therapist (9) Physiotherapist (10) Clinical psychologist (11) Pharmaceutical chemist (12) Phonoaudiologist (13) Dentist (14) Surgical instrument technician (15) Social worker (16) Assistant nursing technician (17) Health technician (18) Other, which one?
	2. Institution where the interviewee works:	Name
	3. How long have you been working in this institution?	__________ years / _______ months
	4. What is the level of healthcare at the institution where you work?	(1) Level I (2) Level II (3) Level III (4) Level IV
	5. Do you work for the state/public sector, private sector, or a public-private institution?	(1) State (2) Private sector (3) Public-private institution
	6. Service where you work	(1) Outpatient service (2) Emergency room (3) Hospitalization (4) Surgical unit (5) ICU (6) Diagnostic service
	7. Which of the following ICT do you use in the patient care process?	a. Desktop computer or laptop b. Tablet c. Internet d. Institutional web page e. Landline or personal mobile phone f. Email g. Electronic medical records h. Electronic patient referral system i. Video conferencing platforms (Zoom, Meet, Teams, etc.)
Attitudes, perceptions, and intention to use		
Perceived usefulness	8. Using healthcare technologies is good for workflow and professional development	(1) Strongly disagree (2) Disagree (3) Neither agree nor disagree (4) Agree (5) Strongly agree (6) DK/NA
	9. I find the use of health technologies helpful for my patients' care (the ability to provide benefits or facilitate certain aspects of care).	
	10. My interaction with healthcare technologies helps me communicate information to my patients.	
	11. Using healthcare technologies allows me to perform tasks quickly.	
	12. Healthcare technologies are tools to improve care, but there are human functions that healthcare technologies cannot perform.	
Perceived ease of use	13. It was easy for me to learn how to use health technologies in the clinical care of my patients.	(1) Strongly disagree (2) Disagree (3) Neither agree nor disagree (4) Agree (5) Strongly agree (6) DK/NA
	14. I find it easy to use health technologies for patient care.	
	15. Using health technologies for care seems like an easy way to interact with my patients.	
Attitude toward use	16. I am willing to continue using healthcare technologies to provide patients with the quality care they need.	(1) Strongly disagree (2) Disagree (3) Neither agree nor disagree (4) Agree (5) Strongly agree (6) DK/NA
	17. I am satisfied when using healthcare technologies for patient care.	
	18. In my opinion, using health technologies can improve the quality of patient care at different levels of care.	
Behavioral intention to use	19. I intend to learn how to use other healthcare technologies for care.	(1) Strongly disagree (2) Disagree (3) Neither agree nor disagree (4) Agree (5) Strongly agree (6) DK/NA
	20. I intend to routinely use other health technologies (other than the usual ones) for care.	
Enabling conditions and organizational factors		
Enabling conditions	21. The institution manager where I work facilitates the use of health technologies for care.	(1) Strongly disagree (2) Disagree (3) Neither agree nor disagree (4) Agree (5) Strongly agree (6) DK/NA
	22. I have the necessary knowledge to use the health technologies available for care in the institution.	
	23. I have the necessary skills to use the health technologies available for care in the institution.	
Organizational factors	24. Does the health institution in which you work provide facilities to access healthcare technologies?	(1) Never (2) Very seldom (3) Sometimes (4) Many times (5) Always (6) DK/NA
	25. Does the institution where you work have strategic or regulatory documents on the use of healthcare technologies?	(1) Yes (2) No
	26. Does the institution where you work have the technological infrastructure necessary to access and use healthcare technologies?	(1) Does not exist (2) Insufficient (3) Sufficient (6) DK/NA
	27. Does the institution where you work have maintenance and technical support staff to help you use healthcare technologies?	(1) Never (2) Very seldom (3) Sometimes (4) Many times (5) Always (6) DK/NA
	28. Have you received training from your institution in the use of health technologies for care?	(1) Yes (2) No

**Table 2:** Table 2. Content validation analysis using Aiken's V coefficient method

Item	Aiken's V				Aiken's V		
	Sufficiency	Clarity	Coherence	Relevance	Item	U	L
1	1.00	1.00	1.00	1.00	1.00	0.92	1.00
2	1.00	1.00	1.00	0.95	0.99	0.90	1.00
3	1.00	1.00	1.00	1.00	1.00	0.92	1.00
4	1.00	0.95	1.00	1.00	0.99	0.90	1.00
5	1.00	1.00	1.00	0.95	0.99	0.90	1.00
6	1.00	1.00	1.00	1.00	1.00	0.92	1.00
7	1.00	0.95	1.00	1.00	0.99	0.90	1.00
8	0.95	0.95	1.00	1.00	0.98	0.88	1.00
9	0.95	0.95	1.00	1.00	0.98	0.88	1.00
10	1.00	1.00	1.00	1.00	1.00	0.92	1.00
11	0.86	0.86	0.86	1.00	0.89	0.75	1.00
12	0.95	0.90	0.90	0.90	0.92	0.79	1.00
13	1.00	1.00	1.00	1.00	1.00	0.92	1.00
14	1.00	1.00	1.00	1.00	1.00	0.92	1.00
15	1.00	1.00	1.00	1.00	1.00	0.92	1.00
16	1.00	1.00	1.00	1.00	1.00	0.92	1.00
17	1.00	1.00	1.00	1.00	1.00	0.92	1.00
18	1.00	0.95	1.00	1.00	0.99	0.90	1.00
19	1.00	1.00	1.00	1.00	1.00	0.92	1.00
20	0.95	0.95	1.00	1.00	0.98	0.88	1.00
21	1.00	1.00	1.00	1.00	1.00	0.92	1.00
22	0.90	0.90	1.00	1.00	0.95	0.84	1.00
23	0.90	0.90	1.00	1.00	0.95	0.84	1.00
24	0.90	0.90	1.00	1.00	0.95	0.84	1.00
25	0.90	0.90	1.00	1.00	0.95	0.84	1.00
26	1.00	1.00	1.00	1.00	1.00	0.92	1.00
27	0.90	0.95	1.00	1.00	0.96	0.86	1.00
28	0.90	1.00	1.00	1.00	0.98	0.88	1.00
Total by dimension	0.97	0.97	0.99	0.99	0.98 (95% CI: 0.88-1.00)		

L: lower limit of interval and in the equation; U: upper limit of confidence interval.

**Agreement among expert judges**

The overall agreement, as assessed by the Brennan-Prediger index, was 0.90 (95% CI: 0.87- 0.93) p= 0.001, representing almost perfect inter-rater agreement. In turn, the inter-rater agreement in the sufficiency category was 0.82 (95% CI: 0.74-0.89), in the clarity category 0.96 (95% CI: 0.92-1.00), in the coherence category 0.95 (95% CI: 0.90-1.00), and the relevance category 0.85 (95% CI: 0.77-0.92), all categories with p-value <0,001. 

Finally, the experts made comments related to the use of terminology in some of the items: "prefer the use of the word manager instead of director," "define in the tool what is useful and good or describe why it is useful or good," and "present examples of video conferencing platforms (Zoom, Teams, Google Meet), and mobile messaging applications (WhatsApp, Telegram, Messenger)." These comments made it possible to adjust and improve the wording of some items 

## Discussion

This study validated the questionnaire using the 1985 Aiken's V coefficient and the adaptation proposed by Penfield and Giacobbi^26^ through expert judgment. The analysis yielded an overall Aiken's V and all item values above 0.80, confirming the instrument's content validity across the evaluated categories. It should be noted that most studies assessing perceptions and attitudes toward technologies do not incorporate an expert judgment validation process prior to statistical validation^15^–^22^. This process is fundamental to assess the conceptual clarity, relevance, pertinence, comprehension, and adequacy of the questionnaire; it helps to identify apparent issues and establishes a strong foundation for the subsequent pilot testing phase^35^. 

On the other hand, healthcare personnel are increasingly exposed to technology in their clinical activities when interacting with patients and performing administrative activities, which requires adapting activities to technological advances^36^. In addition, new digital responsibilities require knowledge, skills, and abilities that staff may not have received the training or support for^37^. The demands in terms of digital competencies, changes related to reduced reliance on paper, shifts in organizational culture, daily use of technologies in practice, and concerns about information security and privacy, among other aspects, affect perceptions and attitudes toward technological tools in daily work^38^. While mastering technologies has the power to maximize digital care, improve service quality, and overcome barriers to service delivery, the effective integration of technologies into organizations depends on the acceptance, perceptions, and positive attitudes of healthcare personnel^13^. 

Regarding the study's limitations, it is important to note that, although the method used provided a quantitative evaluation of the questionnaire items and allowed for review by experts with the necessary training and experience, some degree of subjectivity may still exist in the raters’ interpretation of the questions. Therefore, additional reliability and statistical validity analyses are required as a subsequent step in the validation process to improve measurement accuracy and mitigate possible biases or limitations inherent in the expert evaluation process. 

## Conclusions

The adapted questionnaire achieved adequate content validity, as determined by expert judgment, for assessing perceptions and attitudes toward health technologies. The questionnaire can be considered a useful tool to identify how health personnel perceive the inclusion of health technologies in work settings. The data collected through its application can be used to develop strategies to promote the use of technologies among healthcare workers, thus contributing to their adoption. 
